# Dental Amalgam Exposure and Urinary Mercury Levels in Children: The New England Children’s Amalgam Trial

**DOI:** 10.1289/ehp.10440

**Published:** 2007-11-12

**Authors:** Nancy Nairi Maserejian, Felicia L. Trachtenberg, Susan F. Assmann, Lars Barregard

**Affiliations:** 1 New England Research Institutes, Watertown, Massachusetts, USA; 2 Department of Occupational and Environmental Medicine, Sahlgrenska University Hospital and Academy, Gothenburg, Sweden

**Keywords:** children, dental amalgam, creatinine/urine, environmental exposure, mercury, mercury/urine

## Abstract

**Background:**

Urinary mercury (U-Hg) excretion is a commonly used biomarker for mercury exposure from dental amalgam restorations.

**Objectives:**

Our goal was to determine the most efficient measure of dental amalgam exposure for use in analyses concerning U-Hg in children.

**Methods:**

We analyzed time-sensitive longitudinal amalgam exposure data in children randomized to amalgam restorations (*n* = 267) during the 5-year New England Children’s Amalgam Trial. We calculated 8 measures of amalgam, evaluating current versus cumulative exposure, teeth versus surfaces, and total versus posterior occlusal amalgams. Urine samples collected during follow-up years 3–5 were analyzed for mercury excretion. Multivariate models for current and cumulative U-Hg excretion estimated associations between exposures and U-Hg.

**Results:**

At the end of follow-up, the average (± SD) cumulative exposure was 10.3 ± 6.1 surfaces and 5.7 ± 2.9 teeth ever filled with amalgam, corresponding to 30 ± 21 surface-years. Amalgam measures and U-Hg were moderately correlated. Of amalgam exposure measures, the current total of amalgam surfaces was the most robust predictor of current U-Hg, whereas posterior occlusal surface-years was best for cumulative U-Hg. In multivariate models, each additional amalgam surface present was associated with a 9% increase in current U-Hg, and each additional posterior occlusal surface-year was associated with a 3% increase in cumulative U-Hg excretion (*p* < 0.001).

**Conclusions:**

One single measure of amalgam exposure is insufficient. Studies of cumulative effects of mercury from amalgam exposure in children are likely to have improved validity and precision if time-sensitive amalgam exposure measures are used. In contrast, simple counts of current amalgam fillings are adequate to capture amalgam-related current U-Hg.

Dental caries is the single most common chronic disease of childhood in the United States, and around the world most children have decayed or filled teeth (Diehnelt and Kiyak 2001; Pine et al. 2004; U.S. Department of Health and Human Services 2000). Dental amalgam, which is a mixture of mercury and silver alloy powder, has been used to restore decayed tooth surfaces for > 150 years. Amalgam restorations can discharge elemental inorganic mercury (Hg^0^) into the oral cavity, mostly through vapor, which may then be absorbed into the bloodstream and reach various tissues and organs. A common biomarker for average Hg^0^ exposure is urinary mercury (U-Hg), expressed either as a concentration (micrograms per liter) or excretion (micrograms per hour or micrograms per gram creatinine) (Hansen et al. 2004; Kingman et al. 1998; Morton et al. 2004; Olstad et al. 1987). U-Hg is assumed to reflect the kidney burden of mercury, which accounts for Hg^0^ only; this Hg^0^ results primarily from direct Hg^0^ sources (e.g., dental amalgam discharge) but may also be a product of demethylation of organic (methyl) mercury (MeHg) (e.g., from fish consumption) or other environmental exposures (Barregard 1993; Clarkson et al. 1988; Johnsson et al. 2005). High levels of mercury in the body can be neurotoxic or nephrotoxic, depending on the dose and chemical form. Given the widespread use of amalgam dental restorations in children, concern over the health risks of mercury from amalgam prompted the funding of two randomized clinical trials (Bellinger et al. 2006; DeRouen et al. 2006). These trials consistently found no adverse neuropsychological effects of amalgam in children, but controversy over the safety of amalgam lingers (Fuks 2002; Needleman 2006; Osborne et al. 2002).

Despite concern over the potential hazards of mercury from dental amalgam, little is known about the body burden of mercury resulting from dental amalgam in children. In adults, each additional amalgam surface has been associated with a 5–6% increase in U-Hg excretion, corresponding to an increase of approximately 0.6 μg/g creatinine for an additional 10 amalgam surfaces in the average adult (Barregard 2005; Dye et al. 2005; Kingman et al. 1998). However, the association between dental amalgam and U-Hg may very well be age dependent. Children undergo maturational changes that can substantially affect the absorption, distribution, metabolism, and elimination of chemicals (Bruckner 2000; Scheuplein et al. 2002) as well as potential systemic effects.

An understanding of the specific relationship between dental amalgams and U-Hg excretion in children is important for future studies that aim to investigate U-Hg excretion, either in its direct association with amalgam or using amalgam as a covariate when investigating other exposures of interest. Both current and cumulative U-Hg excretion through time may be relevant for research assessing various adverse health outcomes, and the choice of measure may depend on the specific outcome of interest. For example, current U-Hg may be more relevant for research involving certain renal outcomes that are reversible after ceased exposure, whereas cumulative U-Hg may be more suitable for research involving chronic neuropsychological effects of long-term exposure (Bast-Pettersen et al. 2005; Ellingsen et al. 1993; Grandjean et al. 1999; Kishi et al. 1994).

A particular concern is that the level of amalgam exposure information required to most efficiently and validly predict either current or cumulative U-Hg excretion remains unexamined. Although numerous cross-sectional studies (Dilley and Bawden 1999; Gabrio et al. 2003; Levy et al. 2004; Link et al. 2007; Olstad et al. 1987, 1990; Pesch et al. 2002; Schulte et al. 1994; Suzuki et al. 1993; Trepka et al. 1997) have shown that current U-Hg excretion increases with the presence of dental amalgam in children, these previous studies generally used imprecise measures of amalgam exposure, which may reduce the accuracy of estimates of the association with U-Hg excretion. A prospective study by Khordi-Mood et al. (2001) considered the number of fillings, but U-Hg was measured only 9–12 days after restoration, thereby precluding an investigation of the release of Hg from amalgams in the long term, which is essential for studies of cumulative exposure effects. Most recently, researchers involved in the randomized clinical trial of amalgam in Portuguese children reported a strong, positive association between U-Hg and both the number of amalgam surfaces and time since placement (Woods et al. 2007).

The finding that time since amalgam placement was an important determinant of U-Hg indicates that detailed information on the exact length of time amalgams are in the mouth, expressed as a surface–time measure, may be critical to fully estimate associations with U-Hg at various time points (Bates et al. 2004; Saxe et al. 1999). One small study of North Carolina children attempted to use a surface–month exposure index to quantify each child’s cumulative exposure to amalgam restorations, but only two children of the 21 who provided urine samples had detectable U-Hg levels, precluding statistical evaluation of the association (Dilley and Bawden 1999). The fact that the child with the highest exposure score had the highest U-Hg level suggests that a correlation between these variables may be found in studies including more children with detectable U-Hg (Dilley and Bawden 1999).

In the New England Children’s Amalgam Trial (NECAT), 267 children with no previous amalgam exposure were randomized to receive amalgam restorations as needed for the duration of the 5-year trial. Throughout the trial, NECAT prospectively collected extensive data on the timing and placement of amalgam restorations, thus obtaining updated information on amalgam exposure, including the loss of primary teeth with amalgams. In addition, NECAT collected data on nonamalgam factors that may contribute to U-Hg excretion. Thus, for the first time, this data set allows us to address definitively the measurement question. The objective of the analysis in this paper is to clarify the association between amalgam exposure and U-Hg excretion in children, by creating various indices of amalgam exposure and comparing their ability to predict current U-Hg or cumulative U-Hg excretion through follow-up.

## Methods

### Study participants

NECAT was a randomized clinical trial conducted from 1997 to 2005 to examine the health effects of dental amalgam restorations among 534 children, each followed for approximately 5 years. The details of the study design were previously reported (Children’s Amalgam Trial Study Group 2003), as were the main results of the trial (Bellinger et al. 2006). Briefly, children from two geographic areas (urban Boston, Massachusetts, and rural Farmington, Maine) were recruited if they met the following eligibility criteria: 6–10 years of age at last birthday, no prior amalgam restorations, two or more posterior teeth with occlusal (i.e., biting) surface caries, English-speaking, and no major neuropsychological or renal health disorders. Of 5,116 children screened, 598 met eligibility criteria. Parental consent and child assent were obtained for 534 children, who were then randomly assigned to either amalgam or white resin composite restoration material. The analyses presented in this report include only those children who were assigned to the amalgam treatment group (*n* = 267). The study was approved by the institutional review boards of all participating sites.

### Measurement of dental amalgam exposure

Participants were offered free comprehensive dental care, which included semiannual dental examinations and restoration of caries, for the duration of the 5-year trial. All dental examinations and procedures were conducted at community-based or hospital-affiliated dental clinics by trained NECAT dentists following standardized study protocol. At every examination and treatment visit, pertinent dental data, including the status of each tooth surface and placement of restorations, were documented. The amalgam material used was a dispersed phase amalgam (Dispersalloy; Dentsply/Caulk, Milford, DE, USA).

We calculated eight different methods of measuring dental amalgam exposure to compare current versus cumulative exposure, counts of teeth versus surfaces, and total versus occlusal dentition (Table 1). Surface-years exposure measures, which quantified each child’s net cumulative exposure to amalgam restorations, were calculated as follows: For each surface ever restored with amalgam in the child’s mouth, the total number of years that restoration was present in the mouth (i.e., until exfoliation, extraction, or the end of the trial) was calculated, and these were summed to obtain a cumulative number of surface-years of amalgam exposure per child. We estimated dates of exfoliation as the average of the last dental visit with the primary tooth and the first dental visit with the corresponding permanent tooth. Because dental exams documenting the status of each tooth were performed every 6 months, the date of exfoliation is accurate to within 3 months.

### Measurement of U-Hg

Urine samples were collected at baseline and at each annual follow-up visit. Initially, timed overnight urine samples were collected, but compliance became increasingly problematic, so the protocol was amended mid-trial to collect daytime spot samples at the dental clinic. Samples were sent to a central laboratory (Department of Environmental Medicine, University of Rochester Medical School, Rochester, NY, USA) for analysis. The analytic method is based on the rapid conversion of mercury compounds into atomic mercury suitable for aspiration through the cell of a flameless atomic absorption monitor (Laboratory Data Control Model 1235; Thermo Separation Products, Riviera Beach, FL, USA) (Barber and Wallis 1986; Magos and Clarkson 1972). Biological samples were digested in 45% (weight/volume) NaOH solution in the presence of 1% cysteine.

As a result of increasing the volume of urine analyzed from each child, the detection limit, initially 1.5 μg/L, was reduced to 0.45 μg/L after 1 February 2000. This altered detection limit prevents the direct comparison of U-Hg values from samples taken before and after February 2000. For this reason, only U-Hg data from trial years 3–5 are included in this analysis. Nondetectable concentrations (< 0.45 μg/L) were imputed as 
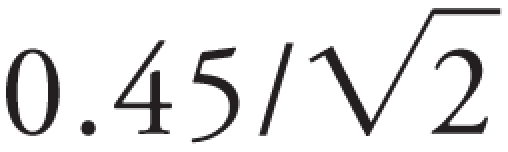
 (Hornung and Reed 1990). Imputation was necessary for 37.9% of U-Hg measures. During the statistical analysis, sensitivity analyses excluded imputed values, and results were similar to the main analysis (data not shown).

Data are presented on the concentration of U-Hg (micrograms per liter) as well as its creatinine-corrected excretion (micrograms per gram creatinine), to allow comparisons with previous studies (1 μg/L = ~ 0.9–1 μg/g creatinine in women and ~ 0.6–0.7 μg/g creatinine in men in overnight urine) (Barber and Wallis 1986; Barregard 1993). We calculated cumulative U-Hg by summing the U-Hg levels obtained at previous visits (i.e., year 4 cumulative U-Hg = year 3 + year 4 current U-Hg; year 5 cumulative U-Hg = year 3 + year 4 + year 5 current U-Hg) (Bast-Pettersen et al. 2005). Overall, we calculated four U-Hg measures: current concentration (micrograms per liter), cumulative concentration (micrograms per liter), creatinine-corrected excretion (micrograms per gram creatinine), and cumulative creatinine-corrected excretion (micrograms per gram creatinine). Because of the skewed nature of the distributions, we used log-transformed values for all U-Hg measures.

### Measurement of potential covariates

During annual study visits, family and sociodemographic data were collected by in-person interviews with parents or guardians, and anthropometric measurements were taken on the children. All interviewers and staff were trained and certified at the New England Research Institutes. The following factors were considered as potential covariates in the predictive models for urinary mercury: age (continuous years), lean body mass (LBM), body weight (kilograms), sex, race/ethnicity (non-Hispanic white, non-Hispanic black, Hispanic, other), socioeconomic status (tertiles of low, medium, or high SES) (Green 1970), frequency of chewing gum (daily, occasionally, not at all), teeth grinding (yes/no), tooth brushing frequency (< 1/day, 1/day, ≥2/day), and fish consumption in both frequency (≥1/week, ≥2/month but < 1/week, ≤1/month, vs. never) and type (total fish, canned tuna, large predatory salt-water fish, other saltwater fish, freshwater fish). LBM was calculated as weight × (1 – percent body fat), with body fat measured by a body fat scale (model TBF-551; Tanita Corporation, Arlington Heights, IL, USA). Assessments for all covariates were updated at each follow-up visit.

Hair samples (50–100 hairs, cut as close to the scalp as possible) were collected at baseline and years 1, 3, and 5 of the trial. We analyzed hair samples for Hg content using a modification of the method in the preceding section on U-Hg measurements; for hair mercury (H-Hg), in the presence of SnCl_2_ (stannous chloride) at high pH, CdCl_2_ (cadmium chloride) breaks the carbon bond, with a subsequent reduction of Hg^++^ to Hg^0^. About half of the hair samples were below detectable concentrations (which vary with hair mass; the average was 0.28 μg/g hair). Nondetectable concentrations were imputed as (detection limit)/√2 (Hornung and Reed 1990), but samples with hair mass < 6.6 mg were excluded from all analyses to limit the inaccuracy that would result from imputing from a very high detection limit. Because H-Hg is considered a biological marker for exposure to organic mercury (e.g., MeHg from fish consumption) (Cernichiari et al. 1995; Morton et al. 2004; Pesch et al. 2002) but MeHg can be demethylated and excreted in urine (Johnsson et al. 2005), H-Hg was considered a potential covariate in the predictive models for U-Hg in a secondary subanalysis including only data from years 3 and 5.

### Statistical analysis

For each of the four measures of U-Hg, we evaluated correlations between the U-Hg measure and each of the eight amalgam exposure measures using Spearman’s rank correlation coefficients to avoid linearity assumptions; results were similar using Pearson correlation coefficients. After evaluating assumptions of linearity, normality, and equal variances, we simultaneously included the three amalgam exposure measures that were most strongly correlated with the U-Hg measure in a repeated-measures regression model. The exposure measure that remained the most statistically significant predictor of U-Hg was selected for use in the multivariate model building, described below.

Repeated-measures analysis of covariance estimated unadjusted and adjusted coefficients for possible predictors of current and cumulative U-Hg, using mixed models with log-transformed creatinine-adjusted U-Hg measures taken at year 3, 4, and 5 follow-up visits. As expected, the distribution of U-Hg was log-normal; we tested model assumptions of normality, equal variance, and the linearity of continuous exposures using residual diagnostics. Factors that were statistically significant (*p* < 0.05) in unadjusted analyses were added to a multivariate model, using a manual stepwise procedure. Factors that remained statistically significant (*p* < 0.05) or changed the estimate of other factors by > 10% remained in the final parsimonious multivariate model. In exploratory analyses, we assessed two-way interactions between amalgam exposure and age or chewing gum frequency in predicting current U-Hg excretion. Because 7–10% of children missed each follow-up visit, and these children differed only in race/ethnicity (more likely to be non-Hispanic black or other race/ethnicity), which was not significantly associated with amalgam exposure or U-Hg, all models assumed missing data were missing completely at random. In sensitivity analysis, we excluded observations with imputed U-Hg (below the detection limit); results were similar in both the magnitude and ranking of correlation coefficients and the magnitude and statistical significance of factors in the multivariate models (data not shown).

In secondary subanalyses, we considered H-Hg as a potential predictor. H-Hg measurements coincided with U-Hg measurements only at years 3 and 5. Because these secondary analyses were restricted to data from two follow-up assessments and samples with sufficient hair mass, the number of observations used (*n* = 302 for current U-Hg; *n* = 210 for cumulative U-Hg) was lower than that in the primary multivariate repeated measures models (*n* = 614 for current U-Hg; *n* = 367 for cumulative U-Hg).

## Results

The average (± SD) age of participants at the first time point used in this analysis (the third annual NECAT exam) was 11.5 ± 1.5 years (range, 8.9–15.6 years). At this time, the mean body weight was 47.6 ± 16.7 kg (range, 22.5–115.7 kg). Half (49.1%) of the children were female. The most common race/ethnicity was non-Hispanic white (61.8%), with 18.4% non-Hispanic black, 5.6% Hispanic, and 14.2% other/mixed race/ethnicity. The children varied in SES, and 27.3% were from families living at or below the federal poverty threshold. Most children (roughly 80%) chewed gum occasionally, whereas < 10% reported chewing gum daily. During follow-up, frequent fish consumption declined, but overall, approximately 27% of children consumed fish at least once per week, and 18% never consumed fish. The most common types of fish consumed were canned tuna or flakey white fish (e.g., halibut, cod), whereas large predatory fish (e.g., swordfish) were least commonly consumed.

Table 1 shows amalgam exposure measured at each follow-up visit. On average, current amalgam exposure decreased during follow-up as a result of the exfoliation of primary teeth that were restored during the first year of the trial. However, most children continued to experience new decay during follow-up, as is evidenced by the increase in mean measures of cumulative exposure. By the end of follow-up, children had on average 30 surface-years of exposure (e.g., 30 surface-years may have resulted from having 10 amalgam-restored surfaces, each present in the mouth for 3 years). Overall, slightly more than half of the filled surfaces were posterior occlusal surfaces.

**Figure 1:**
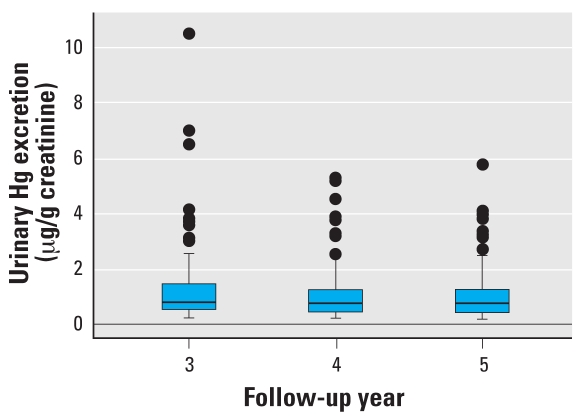
U-Hg excretion during follow-up among children assigned to amalgam restorative treatment in the New England Children’s Amalgam Trial. Boxes indicate upper and lower quartiles, horizontal lines within boxes indicate medians, and error bars indicate 2.5% and 97.5% values with points for outliers.

The mean creatinine-corrected U-Hg excretion was 1.2 μg/g creatinine at year 3, 0.9 μg/g creatinine at year 4, and 0.9 μg/g creatinine at year 5 (Figure 1). Unadjusted for creatinine, the mean U-Hg concentrations were 1.3 μg/L at year 3, 0.9 μg/L at year 4, and 1.0 μg/L at year 5.

**Figure 2:**
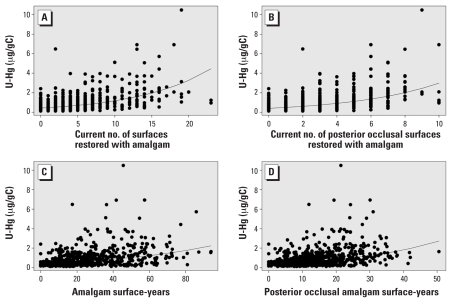
Associations between four amalgam exposure measures and current U-Hg excretion (creatinine-corrected): current number of surfaces (*A*) and posterior occlusal surfaces (*B*), and amalgam (*C*) and posterior occlusal (*D*) surface-years. The regression lines are based on log-transformed U-Hg.

**Figure 3:**
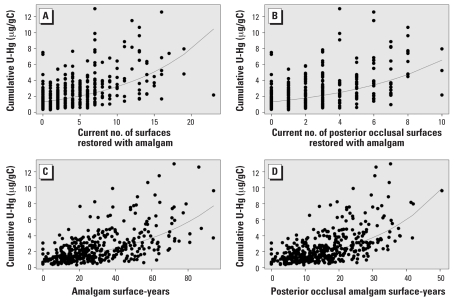
Associations between four amalgam exposure measures and cumulative U-Hg excretion (creatinine-corrected): current number of surfaces (*A*) and posterior occlusal surfaces (*B*), and amalgam (*C*) and posterior occlusal (*D*) surface-years. The regression lines are based on log-transformed cumulative U-Hg.

All amalgam exposure measures and U-Hg measures were statistically significantly correlated (*p* < 0.0001), with moderate correlations (Table 2). The correlations were generally higher for creatinine-corrected U-Hg; for this reason, we used creatinine-corrected measures for the remainder of analyses and in the presentation of results here. To briefly illustrate some of these correlations, Figures 2 and 3 graphically display the correlations between four amalgam exposure measures and current (Figure 2) or cumulative (Figure 3) creatinine-corrected U-Hg excretion. The amalgam exposure measures most correlated with current creatinine-corrected U-Hg excretion were current numbers of teeth, total surfaces, and posterior occlusal surfaces restored with amalgam, with coefficients ranging from 0.53–0.55 (*p* < 0.0001). In a repeated-measures model that included all three of these current exposure measures simultaneously, the only measure that retained statistical significance was current number of surfaces, indicating that it was the most robust predictor of current U-Hg excretion. In contrast, the amalgam exposure measures most correlated with cumulative U-Hg measures were cumulative exposure measures. Interestingly, the cumulative measures using the number of teeth or surfaces ever restored with amalgam were not appreciably better than current numbers. On the other hand, the more specific surface-years exposure measures had notably higher correlations. The posterior occlusal surface-years exposure measure was the most relevant form for cumulative U-Hg (*r* = 0.58, *p* < 0.0001).

The unadjusted and adjusted estimates for the association between potential predictors and creatinine-corrected U-Hg are presented in Table 3. No association was apparent between fish consumption and U-Hg, using either total fish consumption or specific categories of type of fish (data not shown). Statistically significant predictors of current U-Hg in unadjusted analyses were amalgam exposure, age, LBM, body weight, and chewing gum frequency. In building the multivariate model, inclusion of the current number of amalgam-restored surfaces removed the association between age and U-Hg. Similarly, the small but significant association between LBM and U-Hg lost importance when body weight was included in the model, because LBM was correlated with weight (*r* = 0.8, *p* < 0.001) as well as age (*r* = 0.7, *p* < 0.001). On the other hand, chewing gum daily remained a significant predictor of higher U-Hg (*p* = 0.001), and in the presence of amalgam fillings, even occasional gum chewing was associated with higher levels of U-Hg, compared with never chewing gum. In exploratory analyses, there were no statistically significant interactions between current amalgam surfaces and other factors in predicting U-Hg excretion. However, among children who reportedly never chewed gum, the association between amalgam and U-Hg was weaker (β = 0.07, *p* = 0.01) compared with analyses restricted to children who chewed gum daily (β = 0.13, *p* < 0.001; data not shown). Overall, the most important factor in the multivariate model for current U-Hg excretion was the current number of amalgam-restored surfaces, where each additional amalgam surface was associated with a 9% increase in current U-Hg excretion on the original scale (based on log-transformed U-Hg; e.g., e^0.09^ = 1.09), i.e., an increase of about 0.1 μg/g creatinine per surface.

In the multivariate repeated-measures model for cumulative U-Hg (Table 3), the posterior occlusal surface-years exposure measure remained the most statistically significant predictor (*p* < 0.001). Restricting to posterior occlusal surfaces was more efficient than considering all surfaces. As with current U-Hg, cumulative U-Hg excretion was positively associated with daily gum chewing and negatively associated with body weight.

In the secondary subanalysis that considered H-Hg measures taken at years 3 and 5, current H-Hg had a small but statistically significantly correlation with current U-Hg (Pearson *r* = 0.12, *p* = 0.03). In the repeated measures multivariate model (controlled for current amalgam surfaces, weight, and chewing gum), current H-Hg was statistically significant in its association with current U-Hg (β = 0.14, SE = 0.05, *p* = 0.01). Results for cumulative H-Hg (calculated as the sum of H-Hg at years 3 + 5) showed no apparent correlation (unadjusted) between cumulative H-Hg and cumulative U-Hg at year 5 (*r* = 0.11, *p* = 0.11), and in the multivariate model (controlled for posterior occlusal surface years, weight, chewing gum, and follow-up year), there was only a borderline statistically significant association between cumulative H-Hg and U-Hg (β = 0.07, SE = 0.04, *p* = 0.06).

## Discussion

In this longitudinal analysis of various forms of dental amalgam exposure and U-Hg excretion in children, the current number of amalgam surfaces was the most predictive amalgam measure for current U-Hg excretion, whereas the time-sensitive measure of posterior occlusal surface-years of amalgam was most predictive for cumulative U-Hg during follow-up. For all amalgam exposure measures, the correlations were moderate, and they increased when U-Hg concentration was adjusted for creatinine level. Overall, results showed that when modeling U-Hg excretion, the extent of dental amalgam exposure is the most important factor, even among a cohort of children who all have some level of exposure.

These results are consistent with previous cross-sectional studies of the correlation between amalgam and U-Hg in children. For example, a study of 245 German children 8–10 years of age found correlation coefficients of 0.50 for number of amalgam surfaces and 0.49 for number of teeth with U-Hg (micrograms per liter) concentration (Pesch et al. 2002), which is similar to our observed *r* = 0.45. Although the mean U-Hg concentrations in children vary across previous studies (Levy et al. 2004; Olstad et al. 1990; Pesch et al. 2002; Schulte et al. 1994; Suzuki et al. 1993; Trepka et al. 1997), our findings are similar to those of a study of 73 children in Norway, who had an average of 5.8 amalgam surfaces and a U-Hg concentration of 1.0 μg/g creatinine, compared with the NECAT average of 6.2 surfaces and 1.2 μg/g creatinine at year 3 (Olstad et al. 1987). The results presented here generally support the notion that to obtain a rough estimate of current mercury exposure caused by amalgam, it is sufficient to determine the number of filled teeth rather than the number of filled surfaces (Khordi-Mood et al. 2001; Pesch et al. 2002). However, our findings indicate that if more detailed information on surfaces is available, such information improves the accuracy of estimates of the association between amalgam and current U-Hg excretion.

On the other hand, in analyses of cumulative U-Hg, NECAT results suggest that simple counts of the number of amalgams in the mouth are unlikely to capture the full influence of amalgams in children over time. Furthermore, the number of amalgams ever present in the mouth, which can often be easily counted by reviewing dental records, may not suffice. Rather, a more precise measure that takes into account the amount of time each amalgam is in the mouth, such as the surface-years measure, more accurately estimates the association. Such detail may be particularly important in studies of children because restorations in the mouths of children are more likely to have shorter life spans, given the natural course of exfoliation of primary teeth and varying times of eruption for permanent teeth. For studies of U-Hg in relation to central nervous system outcomes such as neuropsychological function, such precision in correlates of cumulative U-Hg may be critical.

In modeling U-Hg excretion, each additional amalgam surface in the mouth was associated with an increase of approximately 9% in current U-Hg excretion. An even greater increase—approximately 20%—was associated with each additional posterior occlusal amalgam surface. These estimates are higher than the 6% increase estimated from studies of adults, a difference that may be attributed to differences in the study populations (Dye et al. 2005; Kingman et al. 1998). Although this sample of children is not directly comparable to the samples of adults, these data provide suggestive evidence that the relative contribution of amalgam fillings to the kidney burden of mercury may be slightly higher in children.

This differential mercury distribution among children may be related to body weight, particularly if fillings in children and adults are of similar sizes, whereby the mercury uptake per kilogram of body weight would be greater among children. The current finding that higher body weight was associated with lower U-Hg excretion supports the importance of body size in the body burden and elimination of Hg^0^ in children. These results are consistent with a previous cross-sectional study that found that children of short height, low weight, and younger age had significantly higher U-Hg excretion (Levy et al. 2004). Given that the sizes of restorations were independent of age and body size, the authors proposed that Hg exposure and subsequent excretion were proportionally greater in younger children because they were smaller. Furthermore, these analyses adjusted U-Hg for creatinine, which “normalizes” the excretion in terms of both body weight (because a large muscle mass will increase the urinary creatinine excretion) and urinary flow rate.

The possibility of a sex difference in U-Hg excretion was recently suggested by a study in Portuguese children, which found that girls excreted significantly higher concentrations of mercury than did boys with comparable amalgam treatment (Woods et al. 2007). We found no statistically significant differences in U-Hg excretion by sex in unadjusted analyses, but a reevaluation of sex in the final multivariate models showed a nonsignificant increase in current or cumulative U-Hg among females (β = 0.07, *p* = 0.38). It is likely that sex-related factors, such as body weight or gum chewing, rather than a genetic sex-related difference in mercury handling alone, may account for much of the sex differences in U-Hg excretion.

The NECAT results confirmed the important role of chewing gum in predicting current U-Hg excretion in children. The impact of chewing on the release of mercury vapor from dental amalgams has previously been observed in studies of adults (Bjorkman and Lind 1992; Sallsten et al. 1996; Vimy and Lorscheider 1985). Accordingly, the finding that the increase in U-Hg was greater for each additional posterior occlusal surface is logical because these surfaces are used for chewing and biting.

Although U-Hg is best known as a marker for inorganic Hg^0^, H-Hg (a marker for organic MeHg) was a statistically significant predictor of current U-Hg excretion. This association is likely attributed to the fact that organic MeHg can undergo demethylation and thereby contribute to urinary excretion of Hg^0^ (Barregard et al. 2006; Johnsson et al. 2005). However, the correlation between H-Hg and U-Hg was small—even weaker than that reported by a previous cross-sectional study of German children (Pesch et al. 2002). Interestingly, none of the measures of fish consumption (the most common source of organic Hg) were associated with U-Hg, suggesting that the measure of H-Hg was a more efficient and valid marker for organic mercury exposure.

A limitation of these analyses was that the change in urine collection protocol midway through NECAT precluded our ability to use U-Hg data from the first 2 years of the trial. For this reason, our analysis of the association between amalgam exposure measures and cumulative U-Hg used data from all 5 years of NECAT for amalgam exposure, but only years 3–5 for U-Hg excretion. A consequence of the inability to use early U-Hg data was that a within-person analysis of change from baseline concurrent with changes in exposure status was not possible. Because children were recruited based on having untreated decay and no prior amalgams, most amalgams were placed at the start of NECAT, and it would have been of interest to compare U-Hg before and soon after the initial amalgam placement. However, with the initial detection limit of 1.5 μg/L at baseline, most children had undetectable concentrations of U-Hg before amalgam placement.

The improved U-Hg detection limit of 0.45 μg/L was still suboptimal, because over one-third of the values required imputation. Although the true effect of omitting the actual values for these imputed measures is unknown, it is reassuring that sensitivity analyses excluding imputed values showed no appreciable differences in our results. Furthermore, given the linearity of the associations between amalgam exposures and detected log-transformed U-Hg values, it is likely that a similar association continued at the lower end of the spectrum of the true undetectable U-Hg values.

This analysis was the first to longitudinally evaluate the associations between various detailed amalgam exposure measures and U-Hg excretion in children. A major strength of this study is the use of repeated measures of exposure and outcome per child, through 3 years of prospective follow-up, including creatinine data to correct for within-person variation in U-Hg measures. The availability of detailed data on the placement, condition, replacement, and extraction of amalgam restorations provided a unique opportunity to investigate the predictive power of amalgam in U-Hg measures. Results indicate that studies of the cumulative effects of mercury in children with varying degrees of exposure to dental amalgam are likely to have improved validity and precision in their analyses if time-sensitive exposure data are used.

## Figures and Tables

**Table 1:** Eight measures of dental amalgam exposure (mean ± SD) at years 3, 4, and 5 among children assigned to amalgam restorative treatment in the New England Children’s Amalgam Trial.

Amalgam restoration exposure measure^a^	Year 3 (*n* = 240)	Year 4 (*n* = 243)	Year 5 (*n* = 248)
Current *n*, teeth^b^	3.5 ± 2.4	2.9 ± 2.3	2.6 ± 2.2
Current *n*, surfaces^b^	6.2 ± 4.9	4.8 ± 4.4	4.1 ± 3.8
Current *n*, posterior occlusal surfaces^b^	3.4 ± 2.3	2.7 ± 2.2	2.4 ± 2.2
Cumulative (ever) *n*, teeth^c^	5.1 ± 2.5	5.4 ± 2.7	5.7 ± 2.9
Cumulative (ever) *n*, surfaces^c^	9.3 ± 5.4	9.8 ± 5.7	10.3 ± 6.1
Cumulative (ever) *n*, posterior occlusal surfaces^c^	4.9 ± 2.4	5.1 ± 2.6	5.5 ± 2.7
Surface-years, total	20 ± 14	25 ± 18	30 ± 21
Surface-years, posterior occlusal	11 ± 6.7	14 ± 8.5	16 ± 10

a *n* = number restored with dental amalgam.

b Current exposure refers to the number present in the mouth at the time of the dental visit. Current exposure measures decreased over time as primary teeth with amalgam restorations exfoliated.

c Cumulative exposure refers to the sum of both current and past amalgams (i.e., including amalgams on teeth that are no longer in the mouth because of exfoliation or extraction).

**Table 2:** Correlation coefficients between eight measures of dental amalgam exposure and U-Hg measures among children assigned to amalgam restorative treatment in the New England Children’s Amalgam Trial (*p* < 0.0001 for all correlations).^a^

	U-Hg concentration (μg/L)		Creatinine-corrected U-Hg (μg/g creatinine)	
Amalgam restoration exposure measure^b^	Current^c^	Cumulative^d^	Current^c^	Cumulative^d^
Current *n*, teeth	0.45	0.47	0.54	0.46
Current *n*, surfaces	0.45	0.47	0.55	0.50
Current *n*, posterior occlusal surfaces	0.45	0.47	0.53	0.45
Cumulative (ever) *n*, teeth	0.33	0.43	0.43	0.51
Cumulative (ever) *n*, surfaces	0.28	0.37	0.38	0.49
Cumulative (ever) *n*, posterior occlusal surfaces	0.32	0.43	0.41	0.50
Surface-years, total	0.30	0.45	0.40	0.57
Surface-years, posterior occlusal	0.32	0.49	0.41	0.58

a Spearman rank correlation coefficient for correlation between amalgam exposure and urinary mercury during annual follow-up visits at years 3, 4, and 5 (*n* = 616). Ranking of correlations for exposure measures was confirmed in sensitivity analyses stratified by follow-up visit (year 3 *n* = 195, year 4 *n* = 204, year 5 *n* = 217).

b *n* = number restored with dental amalgam.

c Measure taken during the same follow-up visit as the current amalgam exposure measure.

d Sum of the individual U-Hg measures taken at each previous follow-up visit. Cumulative U-Hg was calculated for visit 4 (using data from visits 3 and 4) and visit 5 (using data from visits 3, 4, and 5) only.

**Table 3:** Repeated-measures mixed model results for log-transformed creatinine-corrected U-Hg measurements from annual visits at years 3, 4, and 5 of the New England Children’s Amalgam Trial.

	Current U-Hg (μg/g creatinine)^a^				Cumulative U-Hg (μg/g creatinine)^b^			
	Univariate models		Multivariate model		Univariate models		Multivariate model	
Characteristic	β (SE)	*p*-Value	β (SE)	*p*-Value	β (SE)	*p*-Value	β (SE)	*p*-Value
Amalgam surfaces, current *n*^c^	0.10 (0.01)	< 0.001	0.09 (0.01)	< 0.001	0.05 (0.01)	< 0.001		
Posterior occlusal amalgam surfaces, current *n*^c^	0.19 (0.01)	< 0.001			0.09 (0.02)	< 0.001		
Amalgam surface-years	0.01 (0.002)	< 0.001			0.02 (0.002)	< 0.001		
Posterior occlusal amalgam surface-years	0.03 (0.004)	< 0.001			0.05 (0.004)	< 0.001	0.03 (0.004)	< 0.001
Age (years)	–0.12 (0.02)	< 0.001			–0.12 (0.03)	< 0.001		
LBM	–0.01 (0.001)	< 0.001			–0.004 (0.001)	0.004		
Weight (kg)	–0.01 (0.002)	< 0.001	–0.01 (0.002)	< 0.001	–0.01 (0.002)	< 0.001	–0.01 (0.002)	< 0.001
Sex								
Male	0.02 (0.09)	0.82			–0.01 (0.10)	0.90		
Female	reference				reference			
Race/ethnicity								
Non-Hispanic black	–0.04 (0.12)	0.77			0.27 (0.16)	0.08		
Hispanic	0.18 (0.21)	0.39			0.16 (0.23)	0.49		
Other	0.06 (0.15)	0.68			0.02 (0.17)	0.87		
Non-Hispanic white	reference				reference			
SES								
Low	0.08 (0.11)	0.45			0.04 (0.12)	0.72		
Medium	0.06 (0.11)	0.60			–0.004 (0.13)	0.97		
High	reference				reference			
Chewing gum								
Daily	0.44 (0.16)	0.01	0.49 (0.14)	< 0.001	0.30 (0.15)	0.05	0.23 (0.09)	0.01
Occasionally	0.07 (0.12)	0.52	0.19 (0.10)	0.06	0.23 (0.10)	0.03	0.06 (0.06)	0.37
Never	reference		reference		reference		reference	
Toothbrushing frequency								
≥2/day	0.03 (0.15)	0.85			0.11 (0.13)	0.39		
1/day	0.08 (0.15)	0.60			0.01 (0.12)	0.96		
< 1/day	reference				reference			
Grinds teeth (yes vs. no)	0.05 (0.13)	0.70			–0.05 (0.15)	0.76		
Fish consumption^d^								
At least 1/week	–0.20 (0.11)	0.31			–0.15 (0.11)	0.18		
≥2/month	–0.20 (0.11)	0.37			–0.05 (0.10)	0.65		
≤ 1/month	–0.10 (0.10)	0.08			–0.07 (0.08)	0.38		
Never	reference				reference			

a Measure taken during the same follow-up visit as the current amalgam exposure measure.

b Sum of the individual U-Hg measures taken at each previous follow-up visit, and was calculated for visit 4 (using data from visits 3 and 4) and visit 5 (using data from visits 3, 4, and 5) only. Models including age, LBM, or weight were also adjusted for year of follow-up.

c *n* = number restored with dental amalgam.

d Fish consumption categories were mutually exclusive and referred to all types of fish and seafood.
